# Maternal Phenylketonuria: Consequences of Dietary Non-Adherence and Gaps in Preconception Care—A Case Report

**DOI:** 10.3390/jcm14041102

**Published:** 2025-02-09

**Authors:** Julia Donarska, Anna Weronika Szablewska, Jolanta Wierzba

**Affiliations:** 1 Department of Obstetric and Gynaecological Nursing, Institute of Nursing and Midwifery, Medical University of Gdansk, Sklodowskiej-Curie 3A, 80-210 Gdansk, Poland; juliadonarska@gumed.edu.pl; 2 Department of Internal and Pediatric Nursing, Institute of Nursing and Midwifery, Medical University of Gdańsk Sklodowskiej-Curie 3A, 80-210 Gdansk, Poland; jolanta.wierzba@gumed.edu.pl

**Keywords:** phenylketonuria, maternal PKU syndrome, nutritional therapy, newborn defects, pregnancy

## Abstract

**Background:** Maternal phenylketonuria (PKU), a metabolic disorder caused by defective phenylalanine hydroxylase activity, requires strict lifelong dietary management to prevent toxic phenylalanine accumulation. During pregnancy, non-adherence to a low-phenylalanine diet can lead to maternal PKU syndrome, resulting in severe neonatal complications, including microcephaly, congenital heart defects, and growth restrictions. Despite advances in metabolic management and preconception care guidelines, adherence remains a significant challenge, particularly among adults transitioning out of pediatric care. This case report examines the clinical consequences of dietary non-adherence in maternal PKU, highlighting the importance of preconception education, metabolic monitoring, and multidisciplinary care in preventing adverse neonatal outcomes. **Methods:** Using the CARE guidelines, we present the clinical course of a male neonate born to a mother with untreated PKU. **Results:** The analysis incorporates maternal dietary history, prenatal care details, and neonatal outcomes. Additionally, a review of current literature on maternal PKU management and outcomes contextualizes the findings. The neonate, delivered at 38 weeks via cesarean section, exhibited low birth weight (2150 g), severe microcephaly (head circumference: 28 cm), microphthalmia, and septal heart defects. Maternal dietary non-adherence, beginning in late adolescence, contributed to significantly elevated phenylalanine levels during pregnancy (>20 mg/dL). Prenatal care was initiated in the 23rd week of gestation, delaying dietary intervention. The mother reported limited understanding of the teratogenic risks associated with poor dietary control, which was compounded by gaps in preconception counseling and care continuity. **Conclusions:** This case underscores the critical need for comprehensive preconception education and lifelong metabolic management for women with PKU. Early and sustained dietary adherence is essential to mitigate neonatal risks. Public health initiatives should prioritize access to preconception care, enhance patient education, and establish robust multidisciplinary support systems to optimize maternal and neonatal outcomes. Addressing barriers such as delayed care initiation and limited dietary support can significantly reduce the burden of maternal PKU syndrome.

## 1. Introduction

Phenylketonuria (PKU) is an inherited metabolic disorder caused by mutations in the PAH gene, leading to deficient activity of phenylalanine hydroxylase. This enzyme deficiency prevents the conversion of phenylalanine to tyrosine, resulting in elevated phenylalanine levels in the blood. If untreated, high phenylalanine concentrations can cause neurotoxicity, leading to severe cognitive impairment and other neurological complications. Early diagnosis through newborn screening and the implementation of a lifelong low-phenylalanine diet have significantly improved outcomes for individuals with PKU, preventing intellectual disability and other systemic issues [1,2]. Maternal PKU syndrome is a condition that arises when women with PKU have elevated phenylalanine levels during pregnancy, leading to teratogenic effects in the fetus. These effects include severe complications such as microcephaly, congenital heart defects, intrauterine growth restriction, and intellectual disabilities. Ensuring adequate metabolic control through a strict low-phenylalanine diet before and during pregnancy is essential to avoid these risks [3,4,5,6]. These complications significantly contribute to neonatal morbidity and mortality, as well as imposing substantial economic burdens associated with long-term healthcare, developmental support, and lost societal productivity. Poorly managed PKU results in severe health consequences for both mother and child, leading to increased medical expenses for additional care, hospitalizations, and specialized therapies, as well as indirect costs, such as reduced productivity due to caregiving responsibilities. In contrast, early interventions and strict adherence to dietary treatment are more cost-effective, preventing complications such as intellectual disabilities and reducing the overall financial and societal impact of inadequate PKU management [5,7,8].

Despite advances in PKU management, maintaining strict dietary adherence, particularly during pregnancy, remains challenging. In fact, studies suggest that a substantial number of women with PKU struggle to maintain metabolic control during pregnancy, which contributes to a higher incidence of maternal PKU syndrome. According to recent data, up to 40% of women with PKU may experience difficulty adhering to the necessary dietary restrictions during pregnancy despite clear evidence of the detrimental effects of non-adherence [5]. Children born to mothers with poorly managed PKU constitute a particularly vulnerable population, facing lifelong risks of neurodevelopmental impairments, which underscores the importance of achieving strict metabolic control during pregnancy [3,4,5].

A main factor in managing maternal PKU is ensuring that women are well prepared for pregnancy through preconception care and counseling. Transitioning from pediatric to adult care can pose significant challenges for individuals with PKU, as the structure and support system of pediatric care are typically more robust than adult care. This transition has been identified as a period during which many individuals experience difficulties in managing their diet, which is often due to insufficient education or a lack of support [5]. Moreover, the neurocognitive consequences of elevated phenylalanine levels are well established with impacts on both fetal development and long-term neurodevelopmental outcomes. These effects can have profound consequences not only for the child but also for the family and healthcare system. Studies emphasize the importance of preconception dietary training and planning, as well as continuous monitoring of phenylalanine levels throughout pregnancy, to reduce the incidence of maternal PKU syndrome.

The economic burden of rare diseases, including PKU, has been increasingly recognized with the costs of untreated pregnancies contributing to a substantial financial burden on healthcare systems [2,9,10,11,12]. This highlights the need for integrated healthcare strategies that include both clinical care and public health interventions to reduce the risks associated with maternal PKU [1].

Despite these advances, adherence to dietary recommendations remains challenging. Factors such as the restrictive nature of the low-phenylalanine diet, limited access to specialized medical and nutritional care, and gaps in patient education about the risks of suboptimal metabolic control during pregnancy often hinder compliance. Addressing these barriers is important for optimizing maternal and neonatal outcomes and guiding the development of evidence-based clinical practices for women with PKU [12,13,14,15].

While the importance of maternal dietary adherence is well documented, few studies have systematically evaluated the long-term developmental and health outcomes of children exposed to elevated maternal phenylalanine levels in utero. Additionally, the barriers to effective dietary management and their impact on pregnancy outcomes remain underexplored. Addressing these gaps is essential to inform evidence-based clinical guidelines, optimize preconception care, and guide the development of targeted interventions to support women with PKU throughout their reproductive years. This case report seeks to contribute to the existing body of knowledge by illustrating the consequences of maternal non-adherence to dietary management and highlighting the implications for health policy and clinical practice.

## 2. Case Report

This case report aims to describe the clinical presentation, diagnostic findings, and management of a male neonate born to a mother with untreated phenylketonuria (PKU). The report highlights the consequences of maternal non-adherence to dietary management during pregnancy, emphasizing the importance of prenatal metabolic control and postnatal interventions to improve outcomes. The case is structured following the CARE guidelines.

### 2.1. Patient Information

The patient was a male neonate born from the second pregnancy of a 28-year-old woman. The first pregnancy ended in spontaneous miscarriage at six weeks of gestation. The mother was diagnosed with PKU in early childhood and had adhered to a low-phenylalanine diet until the age of 18. Upon reaching adulthood, she discontinued the diet, reporting improved well-being without dietary restrictions. Notably, she reported a lack of awareness about the potential risks of dietary non-adherence during pregnancy. Additionally, the mother was a smoker.

### 2.2. Maternal History

The pregnancy was unplanned, and prenatal care only commenced at 23 weeks of gestation, resulting in challenges in determining the exact gestational age. The mother reported no adherence to a low-phenylalanine diet during pregnancy and received no dietary or metabolic monitoring (did not monitor phenylalanine levels during pregnancy).

Despite our attempts to obtain additional prenatal data, the mother reported that phenylalanine levels were not monitored during pregnancy, and no abnormalities were detected in routine ultrasound scans. Unfortunately, she did not provide imaging reports or additional medical records. Due to the lack of a centralized medical record system, we were unable to verify these findings.

### 2.3. Perinatal Course

The neonate was delivered at an estimated 38 weeks of gestation via cesarean section due to impending fetal asphyxia. Birth weight was 2150 g (small for gestational age), and head circumference was 28 cm (microcephaly). Apgar scores were 9 at 1 min and 10 at 5 min. The neonate was admitted to a tertiary-level neonatal unit on the first day of life for further evaluation.

### 2.4. Clinical Findings

The neonate displayed multiple dysmorphic features and clinical abnormalities, including the following:

- Microcephaly (head circumference below the 3rd percentile)*.
- Microphthalmia and hypotelorism (closely spaced eyes).
- Global increased muscle tone (hypertonia).
- Short lingual frenulum (ankyloglossia).
- Congenital heart defect: ventricular septal defect (VSD).
- Newborn metabolic screening excluded PKU in the infant (phenylalanine level—1.65 mg/dL).
- Diagnostic assessment.

Prenatal exposure to elevated maternal phenylalanine levels due to untreated PKU was identified as the cause of the observed anomalies (the maternal phenylalanine level on the day of delivery was 22 mg/dL, which is over 3–4 times higher than the upper recommended limit for pregnant women: 6 mg/dL or 360 µmol/L). The findings were consistent with maternal PKU syndrome, which is a constellation of teratogenic effects arising from inadequate metabolic control during pregnancy.

Despite the diagnosis of microcephaly, cranial ultrasound performed upon the infant’s admission to the ward revealed no abnormalities. The ultrasound confirmed microcephaly; however, the brain structures were normally developed at the time of assessment: the frontal horn width of the lateral ventricles measured 19 mm, the ventricular system was midline, symmetrical, and not dilated. The choroid plexuses had smooth contours and were not enlarged. The subarachnoid space was not widened, and no pathological findings were observed in the posterior cranial fossa. Conclusion: Cranial ultrasound findings were within normal limits.

### 2.5. Therapeutic Interventions

The healthcare team provided comprehensive education to the mother regarding the irreversible nature of the child’s prenatal damage while emphasizing the potential for improved quality of life through multidisciplinary care. Recommendations included the following:

- Referral to specialists for ongoing management of congenital anomalies and neurodevelopmental support.
- Regular pediatric cardiology follow-up to monitor the VSD.
- Early intervention therapy to address hypertonia and potential developmental delays.

Additionally, the mother received counseling on the importance of dietary management for her health and potential future pregnancies. She was encouraged to consult a dietitian and undergo regular monitoring of phenylalanine levels.

### 2.6. Follow-Up and Outcomes

The neonate was discharged in stable condition with referrals to appropriate specialists for ongoing care. The mother committed to follow-up visits and expressed willingness to engage in dietary management to optimize her health and prevent similar outcomes in future pregnancies.

## 3. Discussion

This case emphasizes the pivotal role of maintaining strict metabolic control in women with phenylketonuria (PKU) both prior to conception and throughout pregnancy to avert maternal PKU syndrome in their offspring. The delayed initiation of prenatal care and poor dietary adherence observed in this case resulted in severe and irreversible neonatal complications. These findings underline the necessity of implementing more robust educational initiatives and support mechanisms tailored to women with PKU, particularly during the transition to adulthood—a period often associated with declining dietary compliance. Addressing these challenges is essential to improving outcomes for both mothers and their children.

Many adolescents and adults with phenylketonuria partially or completely discontinue the low-phenylalanine diet, often prioritizing convenience over health. However, this decision is frequently driven by a lack of awareness about the serious consequences of inadequate metabolic control particularly in the context of maternal phenylketonuria (MPKU) syndrome [12,14]. For instance, the mother described in the case above was unaware of the significant risks associated with poor dietary management during pregnancy, which directly contributed to adverse neonatal outcomes. A cornerstone of MPKU syn-drome prevention is comprehensive preconception education tailored to girls and women with PKU. This education should be led by midwives and gynecologists who play a vital role within the multidisciplinary healthcare team. Midwives are uniquely positioned to enhance patient awareness about the importance of maintaining optimal phenylalanine levels both before and during pregnancy [15,16]. The lack of such educational support in the case described above underscores its crucial impact on pregnancy preparation, its course, and the health of the newborn. European guidelines on PKU recommend that be-ginning at the age of 12, all women with the condition receive regular reproductive and sexual health education. This proactive approach is important for preventing pregnancy complications and safeguarding fetal health. Preconception education should focus on elucidating the teratogenic effects of elevated maternal phenylalanine levels on fetal development while equipping women with the knowledge and tools necessary to mitigate these risks [17,18].

The importance of this education is further underscored by global data published in The Lancet Global Health, which indicates that approximately 48% of pregnancies worldwide, are unplanned. This statistic highlights the need for sexually active women with PKU to adhere to a low-phenylalanine diet even if pregnancy is not immediately planned [19]. In the context of preconception care for women with PKU, one of the midwife’s primary responsibilities is to guide patients toward specialized resources and professionals who can support them in preparing for pregnancy. Referral to a dietitian with expertise in metabolic disorders is particularly essential. A dietitian can provide individualized dietary recommendations to ensure compliance with low-phenylalanine restrictions while also addressing the need for a nutritionally balanced diet. This is crucial for ensuring an adequate supply of essential nutrients vital for fetal development [10,18]. Additionally, during the preconception period, the systematic monitoring of blood phenylalanine levels is of paramount importance. Measurements are recommended at intervals of every two weeks, and both the care providers and the prospective mother should closely monitor the results. Regular assessments enable a timely adjustments to dietary management, ensuring phenylalanine levels remain within the optimal therapeutic range (2–6 mg/dL or 120–360 µmol/L) to minimize fetal complications [10,17]. Maintaining strict dietary adherence during pregnancy presents additional challenges, including nausea, food aversions, social constraints, and the high cost of low-phenylalanine products, all of which can hinder compliance. Non-compliance may lead to increasing the risk of adverse outcomes such as congenital heart defects, microcephaly, and developmental delays in the offspring. Moreover, long-term studies suggest that children exposed to uncontrolled maternal PKU in utero may experience cognitive impairments, behavioral issues, and reduced academic performance; what is more, many adults with PKU demonstrated cognitive impairments, most frequently affecting processing speed, executive function and learning [6].

A significant challenge in the care of women with phenylketonuria (PKU) is the lack of continuity in interdisciplinary support and treatment across different stages of their lives. This gap in comprehensive care can result in severe consequences, as exemplified by the health outcomes of the newborn described above, who was affected by maternal phenylketonuria (MPKU) syndrome [20]. Additionally, some patients deliberately choose not to disclose their PKU diagnosis to members of the healthcare team, including obstetricians and midwives. Such nondisclosure may stem from fears of stigmatization or a lack of understanding regarding the potential health consequences of untreated PKU [21].

In the present case, the mother did not seek obstetric care until the 23rd week of pregnancy, which precluded the initiation of a low-phenylalanine diet at an earlier stage. The absence of dietary management during the critical early period of fetal development contributed to the occurrence of multiple congenital anomalies in the child, resulting in lifelong health implications. This case highlights significant shortcomings in both preconception education and the management of pregnancy in women with PKU. The absence of prenatal ultrasonographic data in this case limits the ability to fully assess the progression of the condition in utero. This underscores the need for improved coordination in prenatal care particularly for pregnancies requiring specialized monitoring. Establishing efficient data-sharing systems and enhancing patient education on the importance of prenatal assessments could improve the early detection and management of similar conditions. This case highlights also the importance of coordinated care and efficient medical data exchange, particularly in pregnancies requiring specialized monitoring. The absence of prenatal phenylalanine level assessments and imaging reports highlights the challenges associated with fragmented healthcare systems and limited health awareness among patients. Strengthening patient education and improving coordination between healthcare providers could enhance early detection and management in similar cases.

To prevent MPKU syndrome and optimize fetal health, dietary intervention should ideally begin prior to conception. However, if this is not achieved, the implementation of a low-phenylalanine diet must commence as early as possible during pregnancy. Maintaining maternal blood phenylalanine levels within the range of 120–360 µmol/L is crucial to minimize teratogenic risks and ensure appropriate fetal development. Frequent monitoring—recommended at least twice weekly—is essential to maintain metabolic control and prevent complications associated with elevated phenylalanine concentrations [5,10,17].

The World Health Organization (WHO) identifies exclusive breastfeeding from birth to 6 months of age as the gold standard for infant nutrition, citing its substantial health benefits for both the child and the mother [22,23]. For infants born to mothers with maternal phenylketonuria (PKU), breastfeeding is recommended, as their metabolic system can typically process the phenylalanine present in breast milk effectively. However, adherence to a strict low-phenylalanine diet by the mother remains crucial to minimizing risks associated with elevated phenylalanine levels in breast milk. In some cases, it may be advisable to monitor the phenylalanine concentrations in the infant’s blood to ensure levels remain within safe limits. This approach underscores the importance of personalized monitoring and dietary management in optimizing outcomes for both mother and child [10,14,24].

If a child is diagnosed with phenylketonuria (PKU), breastfeeding can still be safely practiced but requires meticulous management. The regular monitoring of phenylalanine levels in the child’s blood is essential to tailor feeding strategies to the infant’s individual metabolic needs and ensure safety. In cases where dietary adjustments are insufficient, phenylalanine-free formula may be introduced to supplement or replace breast milk, providing necessary nutrients while maintaining phenylalanine levels within a safe range [24,25].

The patient in question presented with a short lingual frenulum, which was a condition that can potentially hinder effective breastfeeding. However, research suggests that nearly 50% of neonates with this anomaly do not encounter breastfeeding difficulties. When challenges arise, they can often be resolved through intervention from a speech therapist. In such cases, care providers should evaluate the extent to which the shortened frenulum impacts breastfeeding ability. If significant impediments are identified, the patient should be referred promptly to an appropriate specialist, such as a speech therapist or neonatologist, for comprehensive assessment and targeted support [26,27].

By ensuring the continued awareness and understanding of dietary management, healthcare providers can help mitigate risks and support the long-term health of both the mother and future pregnancies [10,17,18]. In the described case, the patient’s mother participated in an educational program, which served as a component of ongoing health support. Research highlights that the most effective educational strategies involve individualized information delivery in a calm and focused environment. To enhance learning, educational sessions should incorporate practical elements, such as interpreting food product labels or identifying online retailers that offer low-phenylalanine products. At the conclusion of each session, the midwife and gynecologist should provide the patient with a summary leaflet or card containing the key points, ensuring that she has a readily accessible reference for future use [17].

Our findings align with previous research emphasizing the necessity of strict dietary adherence in women with maternal PKU syndrome. However, maintaining this regimen during pregnancy remains a challenge due to various physiological and psychosocial barriers. Inadequate adherence can have long-term consequences for both the mother and the child, reinforcing the need for targeted interventions, nutritional support, and patient education programs to improve dietary compliance.

## 4. Conclusions

This case underscores the importance of metabolic control in women with PKU before and during pregnancy to prevent maternal PKU syndrome in the offspring. The delayed initiation of prenatal care and lack of dietary adherence in this case led to significant, irreversible neonatal complications. It also highlights the need for enhanced education and support systems for women with PKU, particularly during the transition to adulthood when dietary adherence often declines. Public health initiatives should focus on improving access to preconception care and metabolic monitoring for women with PKU; enhancing patient education regarding the teratogenic risks of uncontrolled phenylalanine levels during pregnancy; and strengthening multidisciplinary support to ensure adherence to dietary and metabolic management protocols. The case reinforces the need for comprehensive care strategies to support women with PKU throughout their reproductive years, minimizing the risk of maternal PKU syndrome and optimizing neonatal health.

## Data Availability

The original contributions presented in the study are included in the article, further inquiries can be directed to the corresponding author.

## Abbreviations

The following abbreviations are used in this manuscript:

PKU	phenylketonuria
MKU	maternal phenylketonuria
